# LIFE COURSE PATTERNS AND PREDICTORS OF THE RELATIONSHIP BETWEEN ADULT CHILDREN AND THEIR MOTHERS

**DOI:** 10.1093/geroni/igad104.1858

**Published:** 2023-12-21

**Authors:** Mieke Beth Thomeer, Rin Reczek

**Affiliations:** University of Alabama at Birmingham, Birmingham, Alabama, United States; The Ohio State University, Columbus, Ohio, United States

## Abstract

Studies demonstrate that the content and quality of the parent-child tie is highly variable across the life course and that multiple social factors predict different aspects of that relationship. Separate research shows that childbirth experiences (e.g., age at birth) matter for the well-being of both the mother and child. However, limited research considers how the mother’s childbearing history is associated with adult child relationships at mid-life and beyond—namely contact and emotional closeness. We use Sequence Analysis on two linked datasets—the 1979 National Longitudinal Survey of Youth (NLSY79) and the National Longitudinal Survey of Youth-Young Adults (NLSY79YA) (N=1,953) to identify life course patterns of closeness and contact between adult children and their mothers. We identify six unique sequences: (1) mixed quality/contact (9.9%), (2) distant (10.0%), (3) close with decreasing contact (13.2%), (4) increasing emotional closeness with high contact (16.4%), (5) mostly close with high contact (23.2%), and (6) always close with high contact (27.3%). We use regression analysis to estimate how different aspects of the childbearing biography are associated with each category. Relationships characterized by “always close with high contact” were associated with more siblings/childbirths and older age at birth. Relationships associated with “emotionally distance” were associated with births characterized as “mistimed,” being a middle or older child, and younger age at birth. Future analysis will consider selection factors such as family histories. This project demonstrates the need for life course perspectives on the child-mother relationship, recognizing the role of childbearing histories and the important diversity within and between individuals.

